# Postpartum Treatment of a Herniation of the Anterior Uterine Wall due to Remains of Placenta Increta

**DOI:** 10.1155/2018/5921495

**Published:** 2018-10-30

**Authors:** Anis Haddad, Olfa Zoukar, Houda Mhabrich, Awatef Hajjeji, Raja Faleh

**Affiliations:** ^1^Department of Obstetrics and Gynecology, Fattouma Bourguiba Teaching Hospital of Monastir. Rue 1er Juin 1955, 5000 Monastir, Tunisia; ^2^Department of Radiology, Fattouma Bourguiba Teaching Hospital of Monastir. Rue 1er Juin 1955, 5000 Monastir, Tunisia

## Abstract

In recent years, the incidence of placenta accreta and associated complications has increased significantly. The authors report the case of a pregnant woman in the 5th month of pregnancy for premature rupture of the membranes. The placenta was inserted low. The evolution was marked spontaneous work followed by the expulsion of the fetus. The delivery of the placenta was haemorrhagic and incomplete. Ultrasonic testing showed a placental fragment integrated in the thickness of the myometrium. Conservative treatment with methotrexate was published a few days later and MRI showed that the anterior uterine sac was filled with blood clots associated with pelvic effusion. A laparotomy was then performed to resect the pouch and the one-piece fragment. The follow-up was uneventful.

## 1. Introduction

Placenta accreta or abnormally adherent placenta remains a source of concern to any obstetrician despite the progress made in terms of both diagnosis and management during delivery and the postpartum period. Because of the dramatic increase in its incidence in the last few decades, together with the rise in caesarean delivery rates and the still-high maternal morbid-mortality rates, much more vigilance is required. Furthermore, a rescue hysterectomy has a significant psychological impact, mainly because of early permanent loss of fertility. This is why there is a tendency to opt for a conservative treatment whenever possible.

Many authors [1, 2] reported, in isolated cases or limited series, different techniques for uterine preservation. This treatment, whose feasibility and success often remain unpredictable, exposes patients to various morbidities that can pose diagnostic and management problems as was the case we report here. This case consists of an unusual complication of placenta accreta diagnosed in the second trimester of pregnancy and manifested as anterior uterine herniation that was conservatively managed.

## 2. Observation

A 26-year-old woman, fourth parity second gesture two abortion (G4 P1 A2), was referred to our hospital for a 24-hour history of premature rupture of membranes. She was at 22 weeks of gestation with a normal pregnancy. She had a history of prior cesarean section due to severe preeclampsia at 34 weeks of amenorrhea (WA) three years earlier, a spontaneous miscarriage, and a medication-induced termination of pregnancy without complications. Apart from this, she had no other significant past medical history.

**Figure 1:**
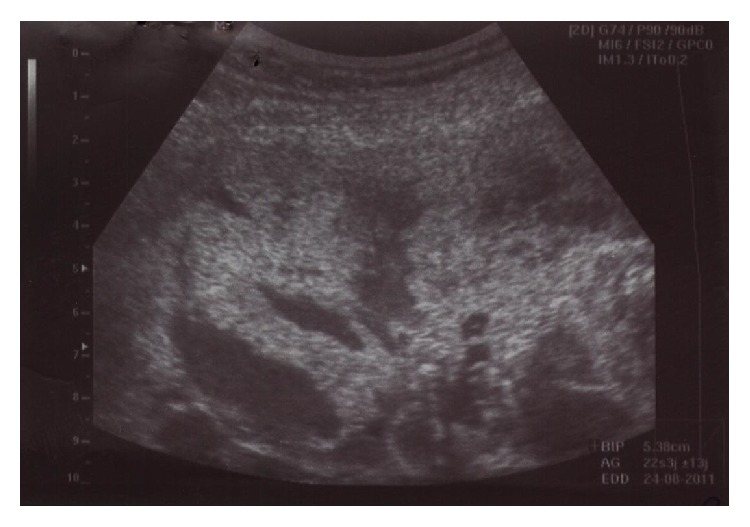
Ultrasonography appearance of a lacunary placenta.

On admission, the clinical examination showed a clear amniotic fluid flow, a spaced out uterine contraction pattern, and a one-centimeter dilated and 50% effaced cervix. An ultrasonographic examination revealed an ongoing viable pregnancy, anamnios and a low-lying anterior placenta with multiple lacunae (Figure 1). The biological findings were suggestive of chorioamnionitis given a CRP at 55.9 mg / ml and a WBC at 16850/ mm^3^ and that was why the prescription of antibiotic therapy was justified.

**Figure 2:**
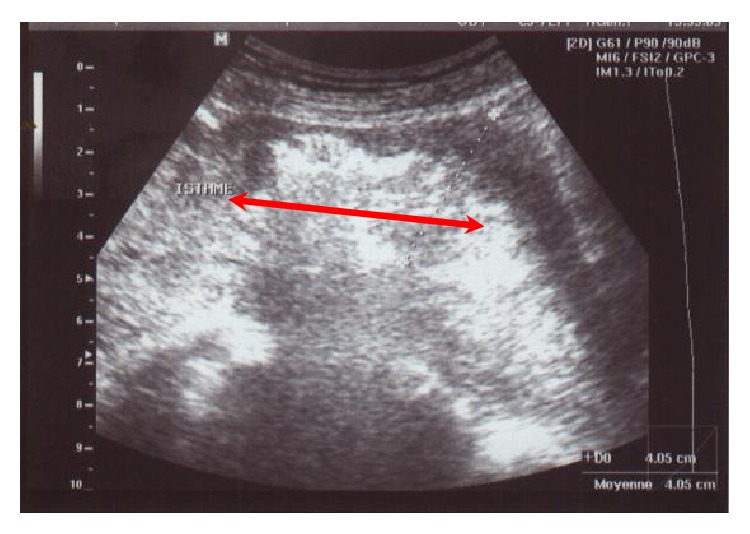
Ultrasonography aspect of the placenta increta fragment.

The evolution was marked by the expulsion of the fetus after 4 hours and the complete retention of the placenta despite an oxytocin infusion already on for 6 hours without any bleeding. The fetal weight was 560 grams.The patient was then transferred to the operating room for uterine revision under general anesthesia. This was difficult and haemorrhagic due to an abnormally adherent placenta. The initial amount of blood lost was about 1200 ml. To control bleeding, a Sulprostone infusion was required in addition to 4 packed red blood cells and 4 fresh frozen plasma bags. An ultrasound performed immediately postabortion revealed only a 3 cm isthmic and echoic image suggestive of a retained placenta increta (Figure 2). Therefore, a medical treatment based on methotrexate was recommended in the absence of bleeding.

**Figure 3:**
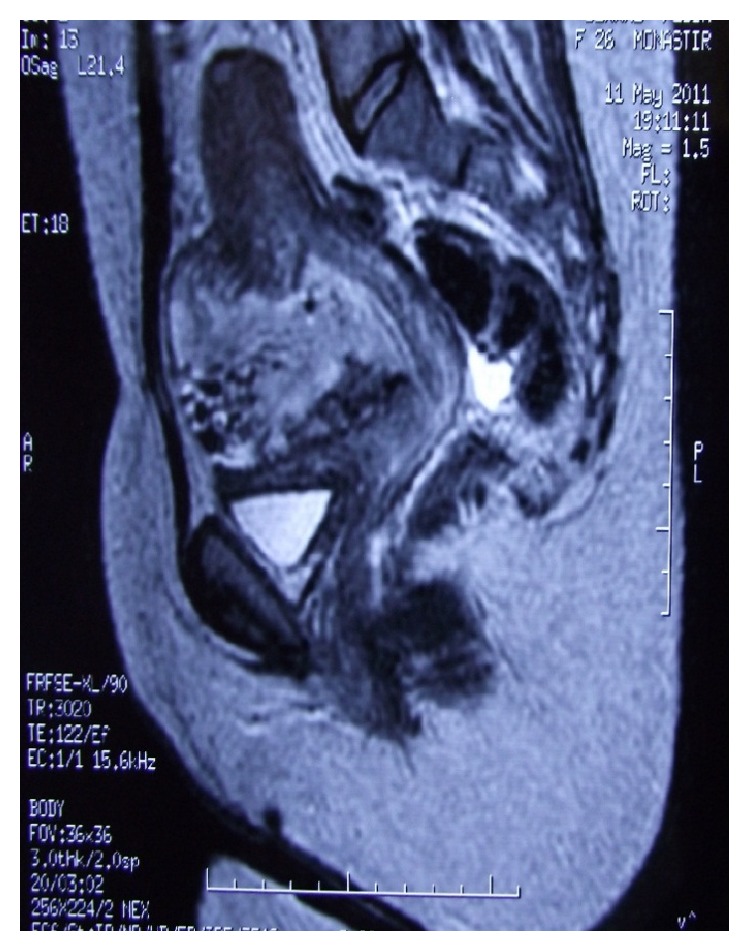
MRI aspect of the anterior uterine sacculation containing the placenta increta fragment with blood clots.

Given the patient's favorable initial evolution, she was discharged on the 5th day with an ultrasound control scheduled in about ten days. The ultrasound showed a swelling of the isthmic region in the form of a hernial sac containing a heterogeneous echoic image of 7 cm along its long axis. There was no associated abdominal effusion. A further exploration by pelvic MRI confirmed previous uterine herniation and revealed a content that was suggestive of an organized hematoma. It also led to a suspected uterine scar dehiscence with possible loss of substance at this level (Figure 3). After the abortion, the placenta remained intrauterine for almost two hours and did not deliver. Accordingly, a laparotomy was decided to resect this sac along with the redundant placenta increta fragment and repair, if possible, this fragile zone. Otherwise, a total hysterectomy would be the ultimate solution.

**Figure 4:**
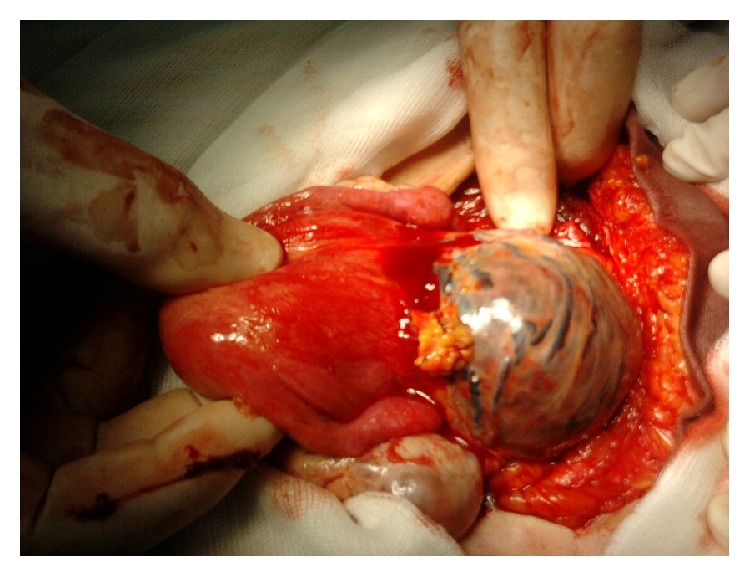
Operative view of anterior uterine sacculation.

**Figure 5:**
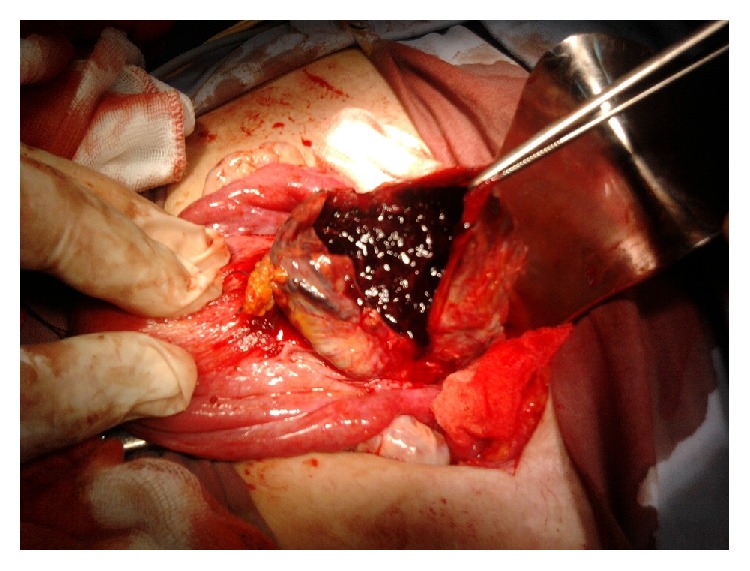
Operative view of the opening of the sac containing placenta increta and blood clots.

**Figure 6:**
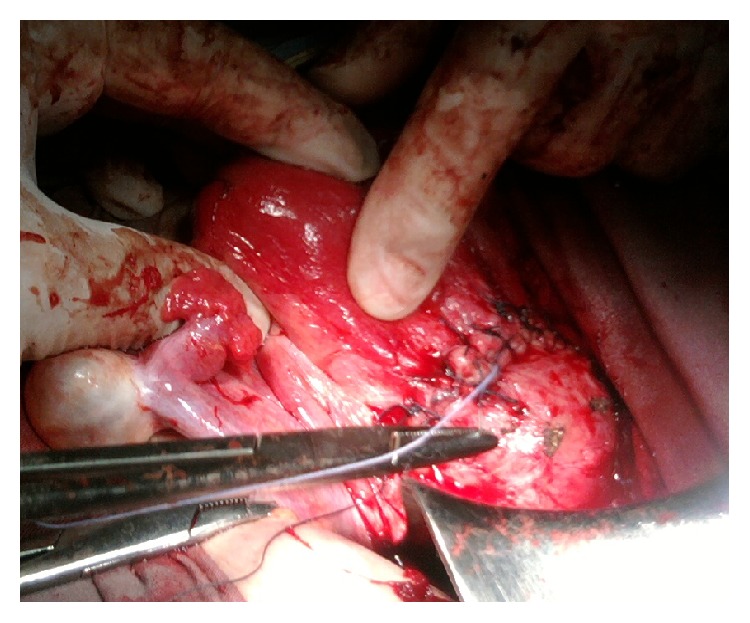
Operative view of the hysterorrhaphy after resection of the sac and its contents.

A surgical exploration revealed an unruptured isthmic hernial pocket, covered by the peritoneum and traversed by multiple dilated veins (Figure 4). Conducting a transverse hysterotomy to open the hernial sac enabled us to easily detach the vesicouterine peritoneum and to evacuate the hematoma (Figure 5). The walls of the sac were then resected along with the increta placental fragments and then a hysterorrhaphy was performed without difficulty by separate points (Figure 6).

Subsequent follow-up was uneventful without any abnormal bleeding. A pelvic ultrasound was normal and there was an insignificant *β*-HCG level. The histopathological examination of the resection specimen confirmed the increta character of the placenta. A hysterosalpingogram performed 6 months later showed a normal uterine cavity without isthmocele.

## 3. Discussion

Our case illustrates well the progressive continuation of a fragment of placenta increta left in place after delivery in the second trimester of pregnancy on a cicatricial uterus despite the medical treatment with methotrexate. This fragment was at the origin of an anterior and isthmic uterine sacculoform neoformation.

In recent decades there has been a rise in the incidence of placenta accreta and its variants (increta and percreta) coinciding with an increasing number of caesarean sections worldwide [3, 4]: currently estimated between 1/2500 [5] and 1/500 [6] deliveries.

Several risk factors are described in the literature, the most important of which are caesarean section scars and curettage [3]. These two factors were present in our patient.

Despite its low incidence, placenta increta continues to be the most feared complication in obstetrics as it is one of the main causes of maternal and fetal/neonatal morbidity and mortality [7, 8].

The ideal management of this complication remains uncertain although much progress has already been made [2]. Ideally, the diagnosis should be made antenatally by medical imaging for women at high risk, which enables health care providers to plan the delivery more effectively and reduce morbidity. Unfortunately, in many cases, especially during the first or second trimester, diagnosis is made only on account of the unusual resistance of the placenta to detachment when attempting a uterine revision [9, 10].

Conventionally, the recommended management consists in a caesarean hysterectomy or hysterectomy scheduled as soon as the diagnosis is retained after the delivery of the fetus, in the presence of a multidisciplinary and experienced team [11]. However, in a recent review of the literature, Rossi et al. found that this procedure, albeit radical, was associated with 53% of maternal morbidity and 3% of maternal mortality [12].

Since the publication of the first case managed conservatively by Arulkumaran et al. in 1986 [13] leaving the placenta in situ and combining chemotherapy with methotrexate as adjuvant therapy, many teams [1, 2, 7, 9] have been performing conservative management preserving the uterus and subsequent fertility. Several methods have been reported about isolated cases or limited series without consensual attitudes [1, 2, 9]. Undoubtedly, this current conservative trend has been facilitated by advances in many parameters: bleeding control by selective embolization and vascular ligation techniques, transfusion of blood and coagulation factors, improved resuscitation, and medical imaging allowing more and more cases of this anomaly to be diagnosed antenatally. In fact, prior awareness of the existence of this pathology allows for better planning and management by bringing together the necessary material and human resources for the smooth running of the treatment.

Although medical imaging is useful for antenatal diagnosis, findings suggestive of placenta increta are not always obvious. In a recent and extensive review of 167 placenta accreta cases, only 44% of them were suspected on ultrasound [8]. In another series, it was only 24% [14]. The diagnosis seems to be more difficult during the early stages of pregnancy, probably due to the paucity of ultrasound signs. Indeed, for Yu M et al. [15], the diagnosis was suspected only in one case among the 31 identified in the second trimester. Although placenta accreta is rare in the 2nd trimester, it is not exceptional. Rashbaum et al. estimated the prevalence of clinical placenta accreta at 0.04% among second trimester abortions [16].

Ultrasound diagnosis of placenta accreta is suspected when there is at least one of the following signs: placental lacunae, obliteration of the retroplacental clear space, interruption of bladder boundaries, andmyometrial thickness less than 1 mm [17]. In our case there was only the first sign and it was interpreted as a subchorionic hematoma.

The MRI is not a first intention in screening, but it is of great help in the presence of technical difficulties with ultrasound (posterior placenta, obese women, etc.) or to make an extra uterine lesional assessment when placenta percreta is suspected [17]. It is the examination of choice for the diagnosis of a uterine herniation while at the same time specifying its anatomical structure and its content [18].

Uterine herniation is a rare and specific complication of pregnancy in which a weakened part of the uterus is transformed into a pouch or hernial sac whose wall contains all the usual uterine layers [19]. Cases reported in the literature include a history of uterine surgery and curettage, uterine malformation, or excessive enzymatic digestion during trophoblast implantation [18, 19]. An association between placenta accreta and uterine herniation has already been suggested [19]. In most cases, the placenta is in the sac and infiltrates the myometrium [19]. The ultrasound diagnosis of the sac is sometimes difficult and remains unknown until delivery, which must be done by caesarean section because of a high risk of uterine rupture [18].

Elsewhere, the retention of a placenta increta fragment is manifested by the delayed onset, in relation to the abortion date, of a heterogeneous echogenic uterine mass. This interval varies from 2 weeks for Ju et al. [20] to 3 years for Lim et al. [21].

This abnormal placentation also predisposes, as described in our observation, to premature delivery and premature rupture of membranes [4, 22]. In the present case, a possible implantation of the egg on the scar of an old caesarean section already weakened by 2 curettages was at the origin of the abnormal invasion of the myometrium and thus the uterine herniation.

Several therapeutic options for conservative treatment are described in the literature [1, 7, 9]. They include surgical treatment and adjuvant therapy. The former can be summarized as the attempt to systematically extract, whenever possible, the maximum of the placenta during delivery or to keep it in situ. Whenever required and when the patient's condition allows, particularly in case of a stable hemodynamic state, a radiological uterine arterial embolization or bilateral vascular ligation of the hypogastric arteries can be helpful [23]. Keeping the placenta in situ seems to be associated with a lower risk of maternal morbidity and secondary hysterectomy, but with a higher predisposition to the risk of sepsis [7].

The adjuvant therapy was based primarily on the administration of a 50 mg/m^2^ body surface area dose of methotrexate to promote placental resorption or its secondary delivery. The median timerequired for complete spontaneous resorption of the placenta was 13.5 weeks (range: 4-60 weeks) in the Sentilhes et al. [8] review. For Timmermans et al., this treatment failed only in 5 out of 22 cases [9].

Overall, Sentilhes et al. found a conservative treatment failure rate of 22%. In these cases a hysterectomy was performed either immediately or secondarily given the extent or recurrence of bleeding or for severe sepsis [8].

In our case, despite the prescription of methotrexate, the placental fragment left in place was a source of endometritis and a progressive increase in the volume of the hernial sac probably due to the pressure exerted by bleeding. An exploratory laparotomy was then carried out on the 18th day for fear of imminent uterine rupture and to control the infection. Indeed the excision of the increta fragment allowed us to repair the weakened uterine zone and to control the infection.

Other authors reoperated on their patients to complete the resection of an evolutive placenta increta fragment by laparotomy [24], hysteroscopy [25], or curettage [26]. Recently Kent et al. [27] demonstrated the feasibility of laparoscopic resection of the sac.

## 4. Conclusion

The trophoblastic invasion of a pregnancy implanted on a cicatricial uterus is a situation that has been on the rise in recent years due to the increase in obstetric scars. This invasion may be the source of an abnormal placentation that causes weakening of the isthmic region, which becomes thin, and an abnormal adherence of the placenta. These abnormalities may in turn be responsible for obstetric morbidity caused by premature rupture of the membranes, late abortion, severe postabortion or postpartum hemorrhage, and placental retention. The retention of a fragment of placenta increta after abortion may remain poorly symptomatic initially and may cause, by an excess of pressure on an already thinned and weakened area, an anterior bulge like a hernia sac. Thus a mass made of placenta and embedded blood is made and can be complicated by infection, rupture, and hemorrhage. The diagnosis is guided by systematic ultrasound which should check, in addition to uterine emptiness, the condition of the lower segment to search in the thickness of the uterine wall of an evocative heterogeneous echogenic image. The MRI is a more precise imaging procedure for identifying herniation and its content. To treat this mass, two approaches can be adopted on a case-by-case basis according to the state of the patient and the radiological workup of the lesion. The first treatment modality is medical, based on methotrexate injections and regular monitoring until the involution of the mass. The second is surgical, aiming at either extracting the increta fragment by curettage or better by hysteroscopy or resecting the hernia and its contents followed by complete reconstruction of the lower segment. It seems that the latter solution is the safest and can be performed by laparotomy or laparoscopy. Arterial embolization may be associated with this treatment.

Conservative management of uterine herniation due to placenta accreta should be considered as the first-line approach for women who desire future fertility. Otherwise, hysterectomy may be the ultimate life-saving solution in case of failure of conservative approaches or if required by the workup of the lesion.
